# Development of energy deposition pixel kernel convolution for planar dosimetry in ^177^Lu therapy

**DOI:** 10.1007/s12149-026-02191-3

**Published:** 2026-03-16

**Authors:** Phornpailin Pairodsantikul, Mizuki Matsushita, Mei Takahashi, Kyeong Min Kim, Hiroshi Watabe, Monchaya Nivorn, Paramest Wongsa, Miho Shidahara

**Affiliations:** 1 Department of Quantum Science and Energy Engineering, Graduate School of Engineering, Tohoku University, 6-6-01-2 Aramaki Aoba, Aoba-ku, Sendai, 9808579 Miyagi Japan; 2 School of Radiological Technology, Faculty of Health Science Technology, Chulabhorn Royal Academy, Bangkok, Thailand; 3 Division of Applied RI, Korea Institute of Radiological and Medical Sciences, Seoul, South Korea; 4 Research Center for Accelerator and Radioisotope Science, Tohoku University, Miyagi, Japan; 5 National Cyclotron and PET Centre, Chulabhorn Hospital, Bangkok, Thailand

**Keywords:** ^177^Lu therapy, Absorbed dose, Energy deposition map, Kernel convolution, Planar Dosimetry

## Abstract

**Background:**

Planar imaging remains widely used for image-based dosimetry in ^177^Lu therapy due to its simplicity and fast acquisition in clinical practice. The only MIRD method is applicable to planar dosimetry. However, conventional MIRD method cannot provide accurate organ-level absorbed dose and patient-specific dose maps. This study develops a novel planar dosimetry method, the energy deposition pixel kernel (EPK) convolution, to improve accuracy of organ dose estimation and provide patient-specific energy deposition maps for planar dosimetry without a high-performance computer.

**Methods:**

EPK convolution generated the energy deposition map by convolving a pixel-based time-integrated activity map with a 2D ^177^Lu energy-deposition kernel incorporating a scaling factor. EPK convolution was validated in simulated two cylindrical water phantoms (containing six hot spheres of varying sizes and two overlapping hot spheres) and two digital human phantoms. EPK convolution was also applied to two open-access ^177^Lu -DOTATATE patients. All absorbed doses estimated from energy deposition maps incorporating partial volume effect (PVE) correction for small spheres and overlap correction (OC) for partial overlap spheres were compared with MC simulation. In case of human phantoms and patients, organ and lesion absorbed doses from patient-specific energy deposition maps incorporating OC and the conventional MIRD method were compared with MC simulation.

**Results:**

Absorbed dose of spheres by EPK convolution showed good agreement with those by MC simulations in both cylindrical phantom studies, with errors within 10% after applying PVE correction or OC. In the digital human phantom and two patient datasets, absorbed doses by EPK convolution were within ± 10% of MC simulation across all organs and lesions, where liver, right kidney and lesions were applied OC. In contrast, the conventional MIRD method showed larger deviations, often exceeding 10% against MC simulation.

**Conclusions:**

EPK convolution provides patient-specific energy deposition maps and improved organ or lesion dose accuracy compared with conventional MIRD method. EPK convolution may contribute for supporting personalized ^177^Lu therapy planning.

**Supplementary Information:**

The online version contains supplementary material available at 10.1007/s12149-026-02191-3.

## Introduction

Radionuclide therapy is a targeted form of internal radiation treatment that delivers therapeutic radiation of radiopharmaceuticals directly to cancer cells or diseased tissue [1, 2]. ^177^Lu -DOTATATE is commonly used radiopharmaceuticals for treating neuroendocrine tumors because ^177^Lu emits beta particles for therapeutic approach and gamma for imaging of post-therapy dosimetry [1–3]. In clinical practice of ^177^Lu -DOTATATE therapy, image-based dosimetry with planar or SPECT/CT images is used to estimate organ and lesion absorbed doses for optimizing treatment outcomes [2–4].

For image-based dosimetry, there are three major methods providing organ-level absorbed dose or voxel-level absorbed dose map [1, 4]. Monte Carlo (MC) simulation is voxel-level approach considered the gold standard due to its accurate calculation of regional energy deposited of each radiation, but it is computationally time-consuming [1, 4, 7]. Medical Internal Radiation Dose (MIRD) method is organ-level approach due to its simple calculation for absorbed doses based on reference phantom but cannot fully account for patient-specific anatomy or provide lesion dosimetry [4, 7]. Kernel convolution is voxel-level approach by convolving 3D-distributions of radionuclide activity with a precomputed dose voxel kernel (DVK) to balance computational efficiency and accuracy. However, kernel convolution is designed for only 3D imaging modalities, not for planar imaging [4–5, 7]. SPECT/CT with multi-bed scans increase acquisition time (30–45 min) and lead to patient burden [5–6]. On the other hand, whole body planar imaging is widely accessible for dosimetry because of simple and fast acquisition in clinical practice. However, MIRD method is currently the only one applied with planar imaging [1, 7].

In this study, we developed a novel 2D kernel convolution method, termed Energy Deposition Pixel Kernel (EPK) convolution, based on the conceptual framework of DVK. While the DVK represents the 3D physics of energy transport, the EPK serves as its 2D projection optimized for planar dosimetry validation. This method aims to improve the accuracy of organ absorbed dose estimation without requiring high-performance computing by providing a patient-specific energy deposition map. This is achieved by convolving a pixel-based time integrated activity (TIA) map with the EPK [8–10], incorporating a scaling factor to correct for variations in object thickness. Unlike conventional MIRD method that rely on standardized phantom geometries, the EPK accounts for pixel-level self- and cross-dose contributions directly from the patient’s planar images. Validation is performed using simulated cylindrical phantoms and digital human phantoms by comparing energy deposition map and absorbed dose value by EPK convolution with MC simulation as a reference. Moreover, to evaluate its potential for clinical application, EPK convolution and conventional MIRD method applies to two open access patient data of ^177^Lu -DOTATATE therapy, and organ absorbed doses are compared with those estimates using MC simulation.

## Materials and methods

### Proposed EPK convolution method

**Fig. 1:**
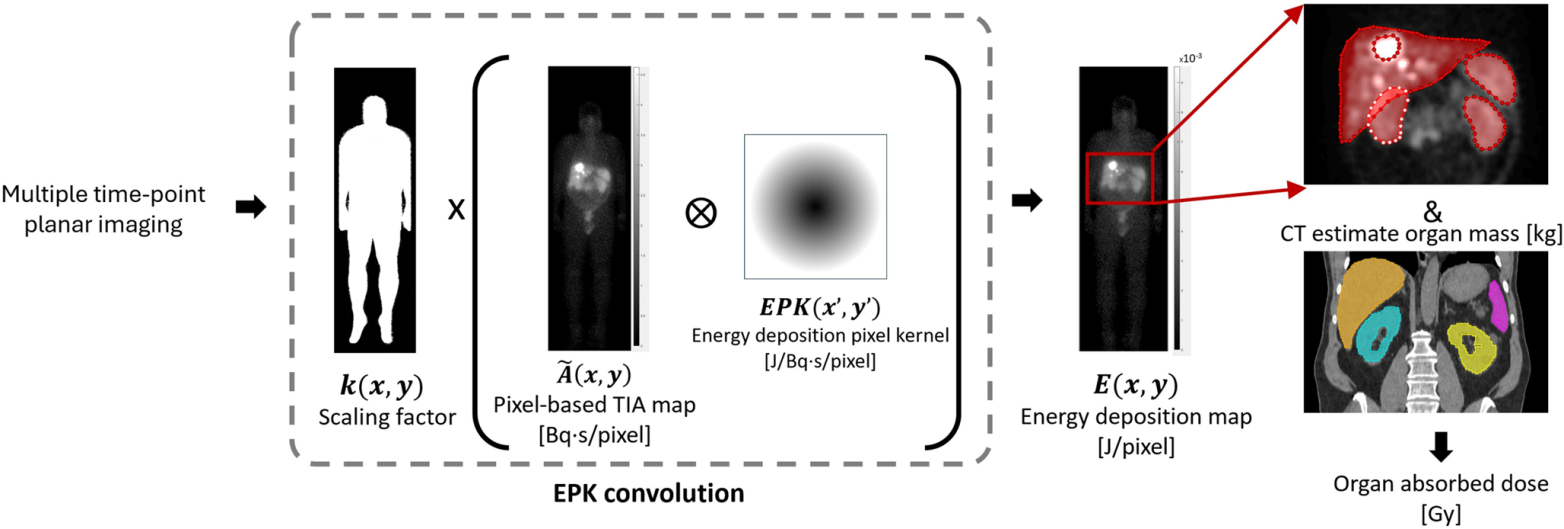
Overview of EPK convolution method and absorbed dose estimation

The proposed EPK convolution method (Fig. 1) estimates energy deposition map $E\left(x,y\right)$ [Joule (J)/pixel] by convolving a pixel-based TIA map $\stackrel{\sim}{A}\left(x,y\right)$ [Bq·s/pixel] derived from time series of planar images with a precomputed 2D energy deposition pixel kernel $EPK\left(x,y\right)$ [J/Bq·s/pixel] as follows:

1 $$E\left(x,y\right)=k(x,y)\cdot(\stackrel{\sim}{A}\left(x,y\right)\otimes EPK\left({x}^{{\prime}},{y}^{{\prime}}\right)),$$

2 $$EPK\left(x,y\right)={\int}_{0}^{z}EVK\left(x,y,z\right)dz,$$

where $\otimes$ indicates the convolution integral, $EPK\left(x,y\right)$ is the 2D projection of an energy deposition voxel kernel of point source, $EVK\left(x,y,z\right)$ [J/Bq·s/voxel]. $k(x,y)$ is a scaling factor, which defined as the ratio of the energy deposition by the corresponding MC simulated (${E\left(x,y\right)}_{MC}$) and EPK convolution $(\stackrel{\sim}{A}\left(x,y\right)\otimes EPK\left({x}^{{\prime}},{y}^{{\prime}}\right))$ to improve the accuracy of absorbed dose calculations as follows:

3 $$\begin{aligned}k\left(x,y\right)&=\frac{{E\left(x,y\right)}_{MC}}{\stackrel{\sim}{A}\left(x,y\right)\otimes EPK\left({x}^{{\prime}},{y}^{{\prime}}\right)}\\&=1-\left(\frac{\stackrel{\sim}{A}\left(x,y\right)\otimes EPK\left({x}^{{\prime}},{y}^{{\prime}}\right)-{E\left(x,y\right)}_{MC}}{\stackrel{\sim}{A}\left(x,y\right)\otimes EPK\left({x}^{{\prime}},{y}^{{\prime}}\right)}\right)\end{aligned}$$

Finally, the absorbed dose [Gy] of organ or lesion can be estimated by dividing the deposited energy of organ or lesion by the corresponding tissue mass (Fig. 1).

### Quantitative planar imaging simulation

The planar images of the cylindrical phantom filled with ^177^Lu solution were generated using the SIMIND MC simulation software V 8.0 (https://simind.blogg.lu.se/) to simulate 5 min images acquisition by a clinical 2 detectors SPECT scanner (GE Discovery NM/CT 670, GE HealthCare Technologies Inc., USA). Only primary radiation at 208 keV (20% energy window) was detected by NaI(Tl) scintillators (40 × 54 cm², thickness of 3/8 inch) with MEGP collimators at a fixed source-to-detector distance of 25 cm [11]. The resulting anterior and posterior planar images, ${C}_{A}(x,y)$ and ${C}_{P}(x,y)$ [count/pixel], are 256 × 256 pixels (2.209 × 2.209 mm²). Planar activity map $A\left(x,y\right)$ [Bq/pixel] was quantified as follows:

4 $$A(x,y)=\sqrt{\frac{{C}_{A}(x,y){C}_{P}(x,y)}{ACF(x,y)}}\cdot\frac{1}{F}$$

where *F* is the conversion factor from count to activity, which is the ratio of total count number and true activity in vial [count/Bq] [1, 12–14]. $ACF(x,y)$ is attenuation correction factor (ACF) defined as ${e^{\mu T}}$, where $\mu$ [cm^-1^] is linear attenuation coefficient and *T* [cm] is the thickness of object. Attenuation correction was performed using a CT-derived ACF, assuming $\mu$ of 0.135 cm^-1^ for ^177^Lu (208 keV) in water [15–16].

### Kernel and scaling factor for EPK convolution

The EPK-kernel in Eq. 2 was generated from $EVK\left(x,y,z\right)$ simulated by PHITS version 3.34 [19]. A cubic water phantom (50 × 50 × 50 cm^3^) with a ^177^Lu point source (1 MBq) at the center was simulated using 100 million histories. The resulting $EVK\left(x,y,z\right)$ [J/Bq·s/voxel] was then projected along the z-axis to obtain a 2D kernel $EPK\left(x,y\right)$ [J/Bq·s/pixel], matching the resolution of the planar images [10, 17–18].

The scaling factor $k(x,y)$ in Eq. 3 was reformulated as a parameter of ACF map $ACF\left(x,y\right)$ as follow:

5 $$\begin{aligned}k\left(x,y\right)&=1-\left(\frac{\stackrel{\sim}{A}\left(x,y\right)\otimes EPK\left({x}^{{\prime}},{y}^{{\prime}}\right)-{E\left(x,y\right)}_{MC}}{\stackrel{\sim}{A}\left(x,y\right)\otimes EPK\left({x}^{{\prime}},{y}^{{\prime}}\right)}\right)\\&=1-[A\cdot\mathrm{ln}\left(ACF\left(x,y\right)\right)+B]\end{aligned}$$

where *A* and *B* are constant derived from fitting the relationship between $ACF\left(x,y\right)$ and the relative error of energy deposition by EPK convolution $(\stackrel{\sim}{A}\left(x,y\right)\otimes EPK\left({x}^{{\prime}},{y}^{{\prime}}\right))$ and the corresponding MC simulation with PHITS software (${E\left(x,y\right)}_{MC}$) [19-21]. The relationship was investigated in 15 radius (5–20 cm) of cylindrical water phantoms (length 30 cm) with both central plane and uniform source of ^177^Lu (37 MBq). To determine *A* and *B* of each source distribution, the relative errors of energy deposition against the corresponding $ACF\left(x,y\right)$ of a 150 rectangular Regions of interest (ROIs) (10 ROIs in each phantom) were fitted by using Eq. 5.

### Validation of EPK convolution in phantom simulation

For the validation of EPK convolution, absorbed dose of ROIs in simulated cylindrical and human digital phantom were compared with those by MC simulation. To improve the accuracy of absorbed dose estimated by EPK convolution, only the partial volume effect (PVE) correction was implemented in simulated cylindrical phantom containing vary size of six hot spheres free from overlapping and only the overlapping correction was implemented in both simulated cylindrical phantom and also digital human phantom containing overlapping spheres or organs and free from PVE [3].

To verify the accuracy of EPK convolution with PVE correction, planar images of the cylindrical cold-water phantom (20 cm diameter and 40 cm length) containing six hot spheres (diameters of 2.0, 3.0, 4.0, 5.0, 6.0, and 7.0 cm, each filled ^177^Lu at 0.282 MBq/ml) placed at the center plane was simulated by SIMIND [22]. Energy deposition map $E\left(x,y\right)$ was calculated using Eqs. 1 and 5 including $\stackrel{\sim}{A}\left(x,y\right)$ assuming physical decay from simulated planar image and $ACF\left(x,y\right)$ of cylindrical water phantom. Six circular ROIs corresponding to each sphere diameter were drawn on the energy deposition map and finally the absorbed dose values [Gy] of each sphere were obtained by dividing total energy deposition [J] with sphere mass [kg]. The absorbed dose values of each sphere corrected for PVE using recovery coefficients (RC) (see Supplemental data 1) were compared to MC simulation results by PHITS [12, 22].

To verify the accuracy of EPK convolution with OC, planar images of the cylindrical cold-water phantom (20 cm diameter and 30 cm length) containing two hot spheres (filled with ^177^Lu in a 2:1 activity ratio, totaling 37 MBq) with diameters of 6.0 cm at coordinates (0, -3, -4) cm and 10.0 cm at coordinates (1, 2, 3) cm was simulated by SIMIND. Simulated planar image resulted in partial overlap of the spheres. Energy deposition map $E\left(x,y\right)$ was calculated using Eqs. 1 and 5 including $\stackrel{\sim}{A}\left(x,y\right)$ assuming physical decay from simulated planar image and $ACF\left(x,y\right)$ of cylindrical water phantom. Two circular ROIs corresponding to each sphere include overlapping area were drawn on the energy deposition map and then total energy deposition was applied OC by subtraction mean energy deposition of involve ROI (see Supplemental data 2). Finally, the absorbed dose values [Gy] of each sphere were obtained by dividing total energy deposition [J] applied OC with sphere mass [kg] and compared to MC simulation results by PHITS.

To evaluate the applicability of EPK convolution to human anatomy, two digital Japanese adult phantoms (male 171 cm 63 kg and female 159 cm 53 kg) [23] were used. Pseudo voxel-based TIA map [Bq·s/ml/voxel] (2 × 2 × 2 mm³, 320 × 160 × 866) for these phantoms were generated by assigning organ TIA values [Bq·s] of ^177^Lu-DOTATATE (from patient A of the open-access SNMMI Dosimetry Challenge dataset [24]) to all voxels of the liver, kidneys, spleen, and remaining body. Energy deposition map $E\left(x,y\right)$ was calculated using Eqs. 1 and 5 including pixel-based TIA map $\stackrel{\sim}{A}\left(x,y\right)$ projected from voxel-based TIA map (see Table S3.1 in Supplemental data 3) and $ACF\left(x,y\right)$ from CT image. Simulated pixel-based TIA map resulted in partial overlap between the liver and the right kidney, and between the liver and the spleen. Three ROIs (liver, kidneys and spleen) were delineated on energy deposition map under CT guidance and total energy deposition of liver and right kidney were applied OC. Finally, the absorbed dose values [Gy] of each organ were obtained by dividing total energy deposition corrected overlapping [J] with organ mass [kg] (by CT image and TotalSegmentator [25] extension in 3D Slicer 5.8.1 (https://www.slicer.org/), see Table S3.3 in Supplemental data3)). Absorbed dose of MIRD method was estimated by MIRDcalc v1.23-Genesis and those of MC simulation was estimated by RTPHITS ver. 1.01.

For each phantom simulation, absorbed dose value by EPK convolution ($${D}_{EPK})$$ (with and without OC) and MIRD method $${(D}_{MIRD})$$ were compared with the corresponding values from MC simulation $${(D}_{MC})$$ as follows:

6 $$\% {\mkern 1mu} {\rm{difference = }}\left( {\frac{{{{\rm{D}}_{{\rm{EPK}}}}\,\,{\rm{ or }}\,\,{{\rm{D}}_{{\rm{MIRD}}}}\,{\rm{ - }}\,{{\rm{D}}_{{\rm{MC}}}}}}{{{{\rm{D}}_{{\rm{MC}}}}}}} \right) \times {\rm{100}}\%$$

### Clinical application of EPK convolution

To assess the clinical applicability of EPK convolution method, two open-access patient datasets of multiple time point SPECT/CT images (256 × 256 × 199, 1.953 × 1.953 × 1.953 mm³) from the SNMMI ^177^Lu-DOTATAE Dosimetry Challenge [26] were used. SPECT/CT imaging of patient A (male, 7.21 GBq) and patient B (female, 7.31 GBq) were performed at 3.7, 27.7, 103.1, 124.0 h and at 3.7, 32.6, 99.6, 193.3 h, respectively. Voxel-based TIA maps were generated using trapezoidal integration with physical decay for the last time-point [10] after registration of SPECT images. Patient-specific energy deposition map $E\left(x,y\right)$ was calculated using Eqs. 1 and 5 including pixel-based TIA map $\stackrel{\sim}{A}\left(x,y\right)$ projected from voxel-based TIA map (see Table S3.2 in Supplemental data 3) and $ACF\left(x,y\right)$ from CT image. Projected pixel-based TIA map resulted in partial overlap of liver and right kidney. ROIs (liver, kidneys, spleen and lesion) were delineated on energy deposition map under CT guidance and total energy deposition of overlap ROIs were corrected the overlapping area. Finally, the absorbed dose values [Gy] of each organ and lesion were obtained by dividing total energy deposition corrected overlapping [J] with mass [kg] (by CT image and TotalSegmentator [25] extension in 3D Slicer, see Table S3.3 in Supplemental data 3). Absorbed dose of MIRD method was estimated by MIRDcalc v1.23-Genesis and those of MC simulation was estimated by RTPHITS ver. 1.01. Then absorbed dose value by EPK convolution (with and without OC) and MIRD method were compared with the corresponding values from MC simulation as shown in Eq. 6.

## Results

### Scaling factor for EPK convolution

**Fig. 2:**
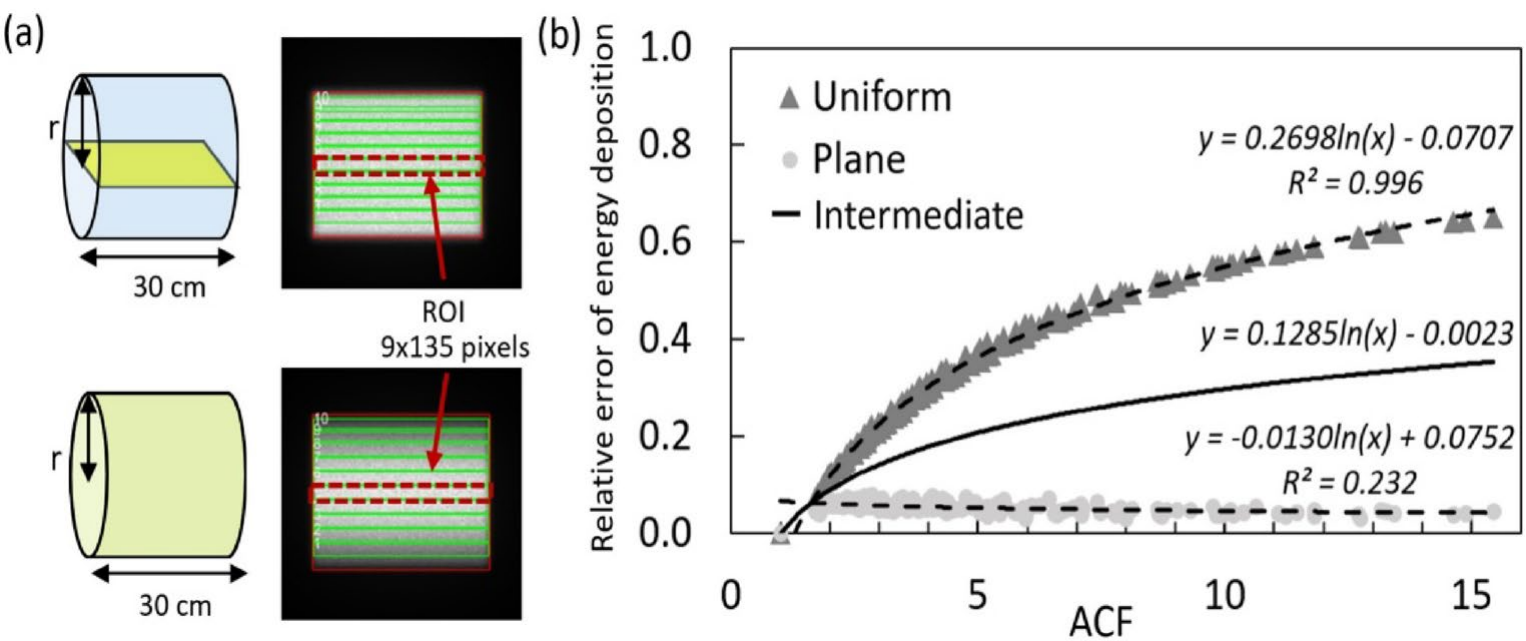
Scaling factor for EPK convolution: (**a**) plane and uniform source distribution in cylindrical water phantom and energy deposition maps by EPK convolution with 10 ROIs for each r and (**b**) relative error of energy deposition against ACF derived from plane, uniform source and the intermediate curve between plane and uniform source

Figure 2 shows the relationship between ACF and the relative error of energy deposition for plane and uniform source distributions in the cylindrical water phantom (Fig. 2(a)). The dependency of source distribution was observed, an intermediate curve between these two extreme cases was established to accommodate various source distributions [20]. The intermediate fitting parameters, A of 0.1285 and B of 0.0023, were used for the scaling factor in Eq. 5.

### Validation of EPK convolution in phantom simulation

**Fig. 3:**
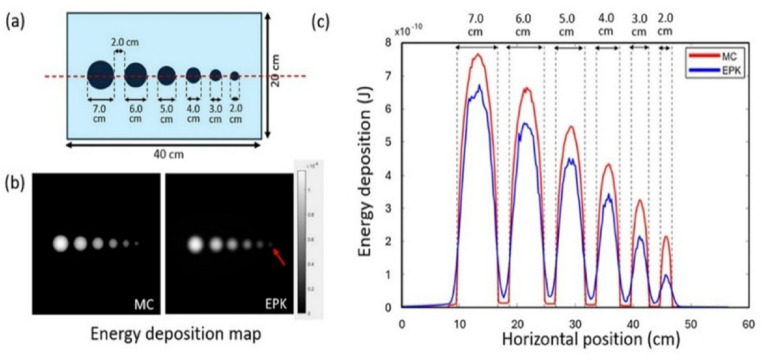
The comparison of (**a**) cylindrical phantom containing six hot spheres about (**b**) energy deposition maps and (**c**) line profile between EPK convolution and MC simulation

Figure 3 shows the comparison of energy deposition maps in the cylindrical cold-water phantom, which contains six hot spheres (Fig. 3(a)) between EPK convolution and MC simulation. In the case of a 2 cm diameter sphere (Fig. 3(b)), EPK convolution tended to be unobservable due to the lower spatial resolution of the imaging system (FWHM = 1.7 cm) [13] , while observable on MC simulation, which directly calculated the distribution of energy deposited in the phantom (independent from imaging system resolution). The spatial resolution of line profile was different between by EPK convolution and MC simulation (Fig. 3(c)).

Table 1 shows the difference of absorbed dose values (in Eq. 6) in each sphere between EPK convolution and MC simulation. The absorbed dose in each sphere by EPK convolution was underestimated due to the PVE, especially in the sphere smaller than 3 cm. When correcting PVE using RC, the absorbed dose by EPK convolution showed good agreement (less than 3%) with that by MC simulation.

**Table 1:** Percentage difference of absorbed dose [%] in six spheres between EPK convolution with RC and MC simulation

Diameter of sphere (cm)	% difference of absorbed dose	
	EPK	EPK&RC
2.0	-38.66	2.91
3.0	-23.65	1.90
4.0	-15.08	1.58
5.0	-8.83	1.82
6.0	-4.14	1.79
7.0	-2.41	1.00

**Fig. 4:**
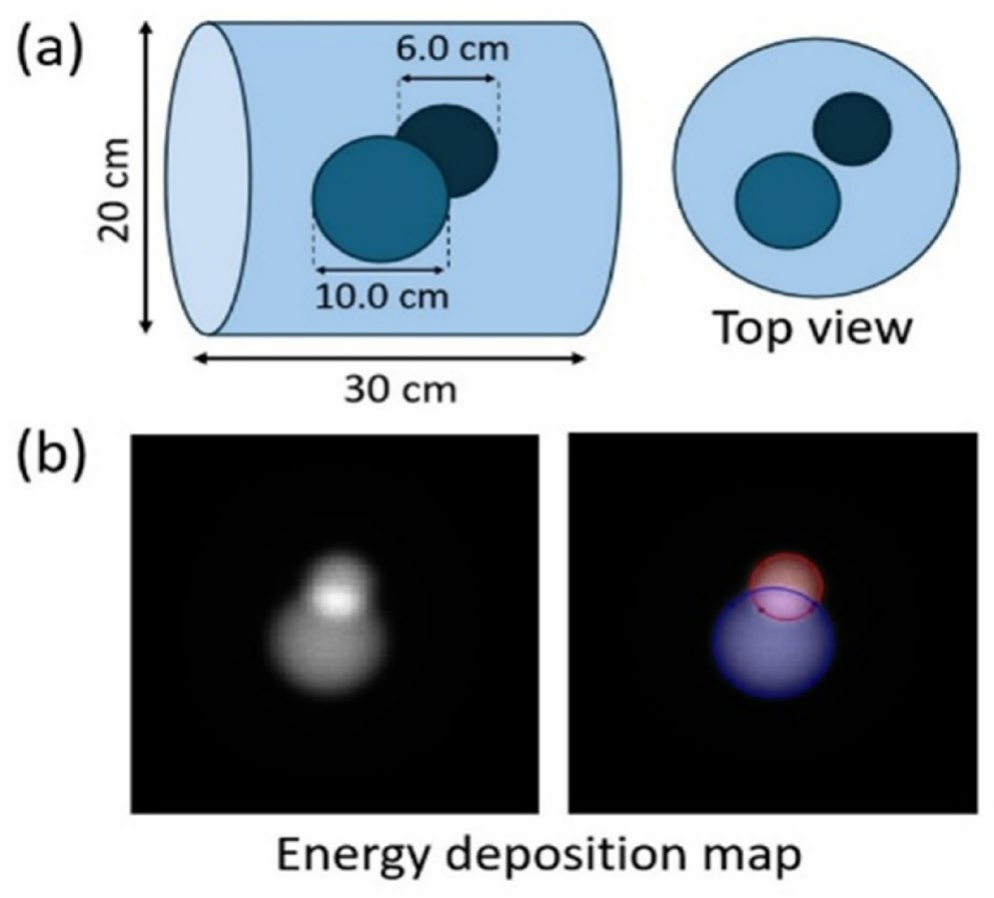
The overlapping spheres phantom in (**a**) 3D geometry and (**b**) energy deposition map by EPK convolution and spheres ROIs

Figure 4 shows energy deposition map of cylindrical phantom containing two hot spheres (Fig. 4(a)), where planar image resulted in partial overlap of the spheres. Higher energy deposition was observed in the overlap region compared with that in non-overlap region (Fig. 4(b)). Table 2 shows the percentage difference of absorbed dose in spheres between EPK convolution and MC simulation. The absorbed dose by EPK convolution in 6 and 10 cm diameter spheres were overestimated 37.90% and 26.88% respectively. After applying OC, the absorbed dose in 6 and 10 cm spheres were reduced 6.92% and 9.76% respectively.

**Table 2:** Percentage difference of absorbed dose in spheres between EPK convolution applied OC and MC simulation

Diameter of sphere (cm)	% difference of absorbed dose	
	EPK	EPK&OC
6.0	37.90	6.92
10.0	26.88	9.76

Table 3 shows the comparison of organ absorbed doses in two digital human phantoms estimated by EPK convolution, MIRD method, and MC simulation. In both phantoms, EPK convolution applied OC showed good agreement with MC simulation across all organs within ± 10% difference. Conversely, EPK convolution without OC exhibited significant deviations, particularly where the right kidney and liver overlapped. In contrast, MIRD method systematically underestimated absorbed doses for all organs ranging from − 13.9% to -43.4% against MC simulation. Additionally, while MIRD provided a single value for both kidneys, the EPK convolution and MC simulation effectively differentiated doses between the right and left kidneys.

**Table 3:** Comparison of organ absorbed dose between 3 methods in two digital human phantoms

Gender	Organ	Absorbed dose (mGy/MBq)				%difference		
		MC	EPK	EPK&OC	MIRD	EPK	EPK&OC	MIRD
Male	Liver	0.54	0.59	0.54	0.31	7.85	-1.28	-43.4
	Rt.Kidney	0.54	0.84	0.57	0.44	57.1	6.48	-16.9
	Lt.Kidney	0.52	0.55	0.55		6.34	6.45	
	Spleen	0.76	0.92	0.82	0.54	20.7	7.30	-29.4
Female	Liver	0.59	0.66	0.61	0.40	11.8	3.04	-32.8
	Rt.Kidney	0.61	0.72	0.63	0.52	18.4	3.43	-13.9
	Lt.Kidney	0.59	0.57	0.57		-3.72	-3.75	
	Spleen	0.83	1.09	0.78	0.65	30.8	-6.31	-22.1

### Clinical application of EPK convolution

**Fig. 5:**
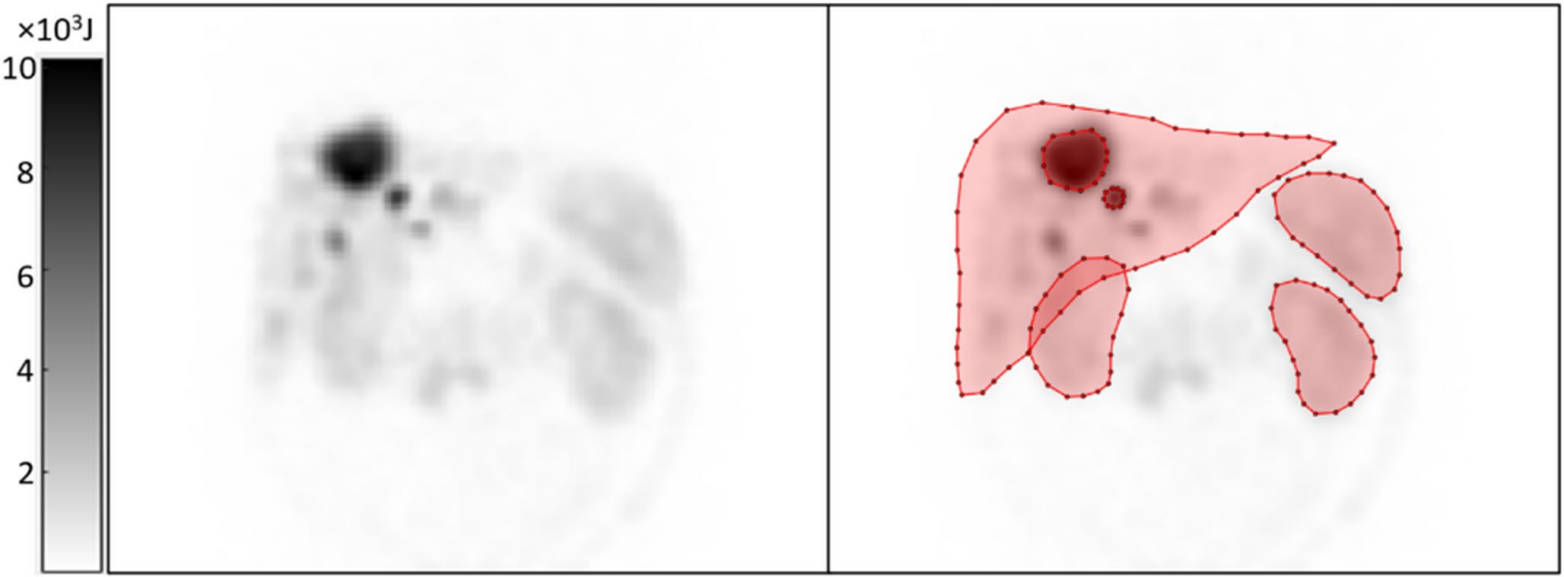
Patient-specific energy deposition map of patient A treated with ^177^Lu-DOTATATE by EPK convolution and ROIs

Figure 5 shows patient-specific energy deposition map of patient A treated with ^177^Lu-DOTATATE by EPK convolution and ROIs. For both patients, the absorbed dose values by EPK convolution applying OC showed good agreement with MC simulation across all organs and lesions within ± 10% difference (Table 4). Conversely, EPK convolution without OC exhibited substantial overestimations in overlapping regions, particularly for the liver and right kidney, as well as lesions located within the liver. In contrast, MIRD method systematically overestimated absorbed doses for all organs ranging from 10.8% to 33.0% against MC simulation (Table 4).

**Table 4:** Comparison of organ absorbed dose between 3 methods in patient data

Patient	Organ	Absorbed dose (mGy/MBq)				%difference		
		MC	EPK	EPK&OC	MIRD	EPK	EPK&OC	MIRD
A (male)	Liver	0.48	0.65	0.46	0.61	34.37	-5.39	26.6
	Rt.Kidney	0.65	0.84	0.66	0.92	29.09	2.78	33.0
	Lt.Kidney	0.73	0.80	0.80		8.97	9.00	
	Spleen	0.90	0.92	0.92	1.16	1.96	1.98	28.5
	Lesion 1	2.51	3.14	2.71	-	25.25	8.27	-
	Lesion 2	2.11	3.68	2.27	-	74.36	7.66	-
B (female)	Liver	0.26	0.32	0.28	0.29	20.29	6.90	10.8
	Rt.Kidney	0.71	1.34	0.76	0.85	89.04	6.66	31.1
	Lt.Kidney	0.58	0.63	0.63		8.72	8.72	
	Spleen*	-	-	-	-	-	-	-
	Lesion 1	0.51	2.66	0.53	-	425.29	5.10	-
	Lesion 2	1.55	2.95	1.67	-	90.26	8.08	-
	Lesion 3	0.52	0.74	0.54	-	43.50	5.21	-
	Lesion 4	0.53	4.52	0.57	-	759.26	7.68	-

^*^Spleen was removed by surgery in patient B

## Discussion

In this study, the novel 2D kernel convolution termed EPK convolution was developed for practical absorbed dose estimation using planar images in ^177^Lu therapy based on the concept of DVK convolution. The validation of EPK convolution in phantom simulation suggested the accuracy within 10% of absorbed dose values with RC and OC (Tables 1, 2 and 3). We also applied EPK convolution to a clinical study, the accuracy of absorbed doses in organ and lesion were within ± 10% compared by MC simulation (Table 4).

Energy deposition pixel kernel, $EPK\left(x,y\right)$, in Eq. 2 was designed to suppress the depth information of DVK. In this study, spatial coverage of 50 × 50 × 50 cm^3^ for EPK was referred to typical planar imaging field of view and encompassed both range of beta emissions (< 1 cm) and gamma emissions of ^177^Lu. Previous studies on DVK for ^177^Lu [17-18] have shown that while beta particles contribute to highly localized deposition, gamma emissions result in broader energy spread. Thus, kernel size should be sufficiently large 50 cm to cover the energy distribution throughout the entire image. Furthermore, the pixel size of EPK should match with pixel-based TIA map, EPK was adjusted pixel size in each simulation of this study to ensure accurate spatial alignment during convolution, consistent with previous study [9–10].

The scaling factor $k\left(x,y\right)$ in Eq. 5 was introduced to improve the accuracy of energy deposition map and absorbed dose value in EPK convolution. The formulized scaling factor succeeded in representing the relationship between relative error of energy deposition against ACF (Fig. 2(b)), however, the dependency of source geometry was observed. An intermediate curve between these two extreme cases was used for scaling factor $k\left(x,y\right)$. As a result, validation using a phantom containing six hot spheres showed that EPK convolution with RC was good agreement with MC simulation (less than 3%, Table 1).

RC and OC were also introduced to improve accuracy of absorbed dose value by EPK convolution because of low spatial resolution and partial overlapping organs in planar image. As shown in Tables 1 and 2 demonstrated the impact of RC and OC for EPK convolution. Tables 3 and 4 demonstrated the importance of the combination of EPK and OC. For practical implementation of EPK convolution, other image-based correction techniques of PVE and overlapping region can also be applied together [27–29].

The application in both human digital phantom and patients (Tables 3 and 4) suggested that EPK convolution, which provides the patient-specific energy deposition map (Fig. 5), is promising for the future clinical planar dosimetry in^177^Lu therapy rather than conventional MIRD method. Absorbed dose by MIRD method were more than 10% different from MC simulation due to discrepancy of organ mass and anatomy between human digital phantom/ patient and reference model in the MIRDcalc software [30] (see Table S3.2 in Supplemental data 3). Although the patient-specific S-values [31] improved organ-level dose but it assumes uniform distribution and doesn’t provide the patient-specific energy deposition map. EPK convolution accounts for pixel-level self- and cross-dose contributions without organ segmentation. Moreover, EPK convolution can visualize organ dose heterogeneity, enabling a more detailed and flexible framework for personalized planar dosimetry with fast computation time. While the reference RT-PHITS MC simulations required approximately 30 h per patient to achieve statistical convergence with 3.0 × 10^7^ histories, the EPK convolution was performed in under 1 s per patient, performed on a computer equipped with an Intel Core i7-12700 K CPU running at 3.6 GHz and 128 GB of RAM, running Windows 11.

In this study of human digital phantoms and patients, EPK convolution used an ideal pixel-based TIA map [Bq·s/pixel], which is projection of voxel-based TIA map [Bq·s/voxel] from quantitative 3D-SPECT image, to exclude the uncertainty of quantitative planar for validation and organ mass estimation from the CT image in the same acquisition with the SPECT image. However, to apply EPK convolution in clinical practice, it is necessary to consider the uncertainty of quantitative planar images such as attenuation correction, scatter correction and calibration factor [32–33]. Additionally, the acquisition date of the CT imaging used to estimate organ mass must be considered to minimize variations in body weight and organ dimensions. In scenarios where significant anatomical shifts or shape differences occur due to varied patient positioning, the application of deformable image registration can be applied to align the CT-based anatomy with the SPECT or planar geometry [34]. Implementing registration techniques is a prerequisite to ensure that the overlapping correction and pixel-level dose conversion are performed on anatomically consistent regions. Further clinical studies involving larger patient groups and calibration of quantitative planar are warranted to validate the robustness, reproducibility, and generalizability of EPK convolution across diverse anatomical and imaging scanners.

## Conclusion

EPK convolution succeeded in providing patient-specific energy deposition maps. The combination of EPK convolution with RC or OC improved accuracy of absorbed dose estimation compared with conventional MIRD method. EPK convolution may contribute for supporting personalized ^177^Lu therapy planning.

## Supplementary Information

Below is the link to the electronic supplementary material.

Supplementary Material 1

Supplementary Material 2

Supplementary Material 3
