# Supplementary material for: Development of energy deposition pixel kernel convolution for planar dosimetry in 177Lu therapy
Source: Ann Nucl Med. 2026 Mar 16;40(7):814–22. doi: 10.1007/s12149-026-02191-3 (PMC13283131; doi:10.1007/s12149-026-02191-3)
Supplement: Supplementary file 1 — Supplementary Material 1 [file 12149_2026_2191_MOESM1_ESM.docx]

**Supplemental Data 1**

**Partial volume effect correction**

Due to the partial volume effect (PVE) in small volumes resulting in underestimation of activity concentration and consequently absorbed dose, partial volume correction was applied to the regional absorbed dose estimation using the recovery coefficient (RC) method. To evaluate the impact of PVE and the effectiveness of RC correction, a cylindrical phantom with a diameter of 20 cm was used, containing five spheres with diameters of 2.0, 3.0, 4.0, 5.0, 6.0 and 7.0 cm positioned centrally. The spheres were filled with activity concentration of 0.45 MBq/mL [1,2]. The recovery coefficient was defined as the ratio between the measured activity concentration on the image and the known true activity concentration. The RC calibration curve was generated by fitting RC values across different sphere sizes using the sigmoid function as:

$RC=\frac{a}{1+b\cdot e^{-c\cdot D}}$ (S1.1)

where *a*, *b* and *c* are the three fit parameters and *D* is sphere diameter in cm.


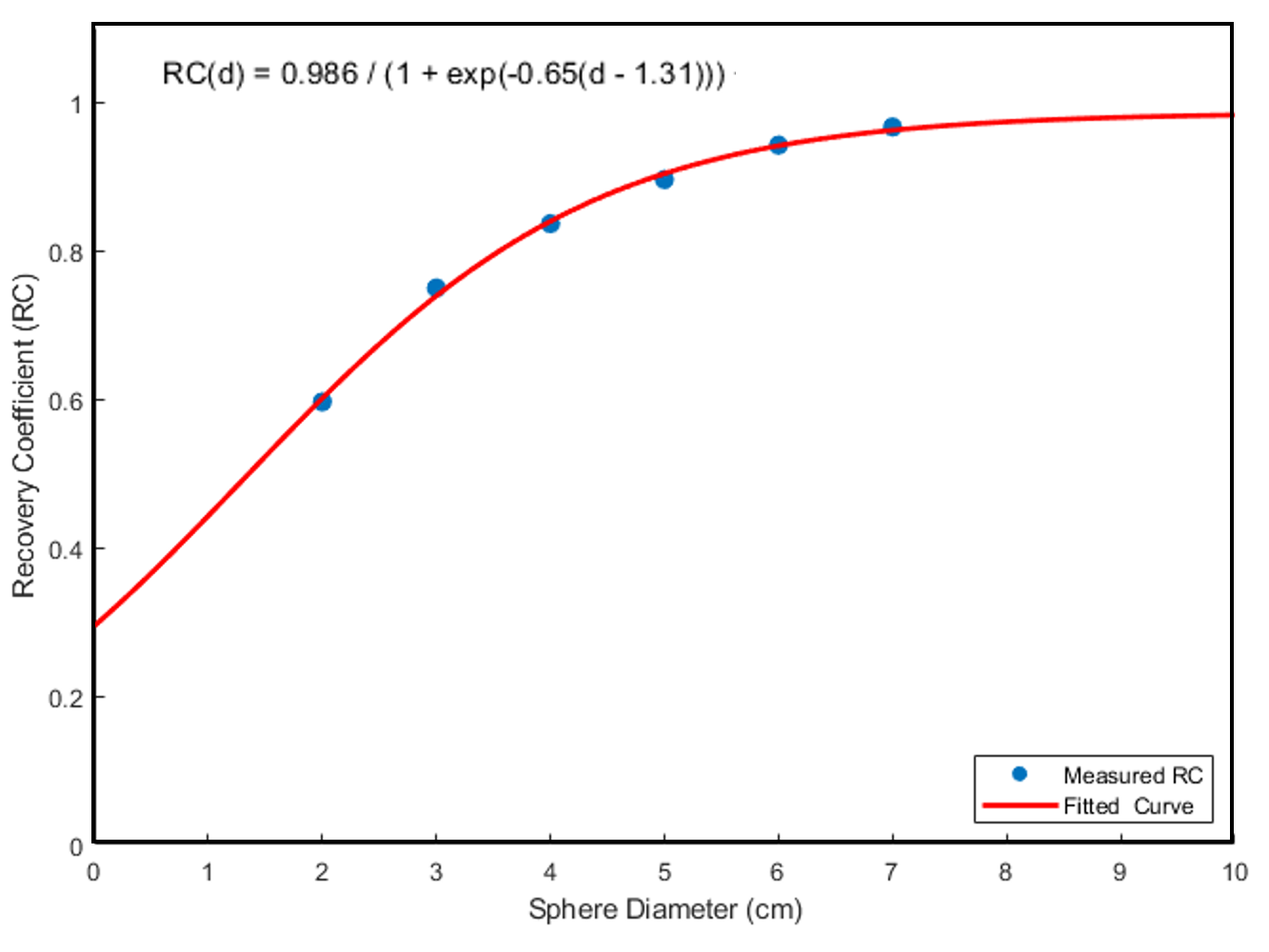


**FIGURE S1.1** Plots of RC as a function of sphere diameter

**Reference**

1. Miller C, Filipow L, Jackson S. A review of activity quantification by planar imaging methods. J Nucl Med Technol. 1995;23(1):3-9.
2. Liu Y, Lu Z, Chen G, Shi K, Mok GSP. Partial volume correction for 177Lu-PSMA SPECT. EJNMMI Phys. 2024;11(1):93. doi:10.1186/s40658-024-00697-1.
