# Supplementary material for: Development of energy deposition pixel kernel convolution for planar dosimetry in 177Lu therapy
Source: Ann Nucl Med. 2026 Mar 16;40(7):814–22. doi: 10.1007/s12149-026-02191-3 (PMC13283131; doi:10.1007/s12149-026-02191-3)
Supplement: Supplementary file 2 — Supplementary Material 2 [file 12149_2026_2191_MOESM2_ESM.docx]

**Supplemental Data 2**

**Overlapping correction**

Organ overlaps in planar imaging leads to artificially elevated intensity values in the overlapping regions, it arises from the projection of 3D anatomical structures onto 2D plane leads to overlapping shadows of organs. As illustrated in Fig.S2.1, the overlapping area between ROI_A_ and ROI_B_ exhibits higher intensity than the surrounding regions due to this effect.


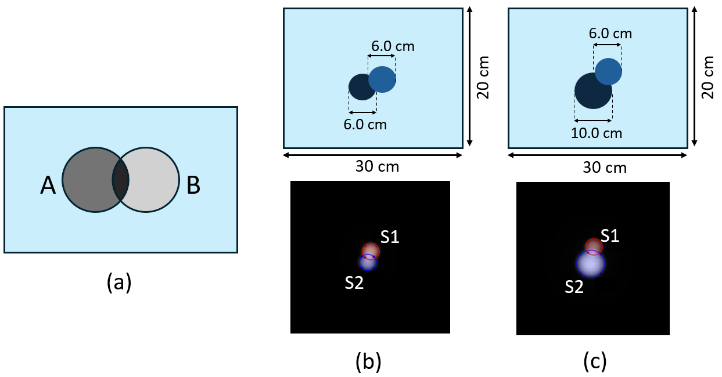


**FIGURE S2.1** Overlapping of ROI_A_ and ROI_B_

Such overlaps can complicate both diagnostic interpretation and quantitative analysis. To address this issue in the energy deposition map, subtraction of energy deposition was applied to correct for the overlapping region [1]. The corrected total energy deposition for ROI_A_ ( $\sum_{i=1}^{n} A$) was calculated as:

$\sum_{i=1}^{n} A={\sum_{i=1}^{m} A}_{i,A-(A\cap B)}+{\sum_{i=1}^{n-m} (A}_{i,(A\cap B)}-\bar{B}_{i,B-\left( A\cap B \right)})$ (S2.1)

Where $A\cap B$ denotes the overlapping region between ROI_A_ and ROI_B_, ${\sum_{i=1}^{m} A}_{i,A-(A\cap B)}$ is the total energy deposition in ROI_A_ excluding the overlapping region, $\bar{B}_{B-\left( A\cap B \right)}$ is the mean energy deposition in the non-overlapping part of ROI_B_, $A_{(A\cap B)}$ is the energy deposition in the overlapping region and $N_{A\cap B}$ is number of pixels within the overlapping region. The same correction approach is applied reciprocally to ROI_B_. This overlapping correction (OC) method ensures that contributions from overlapping regions are correctly partitioned among the involved ROIs, thereby improving the accuracy of energy deposition and absorbed dose estimation. For scenarios involving three or more overlapping tissues, the framework iteratively subtracts the estimated contributions of all other intersecting organs based on their respective mean energy depositions in non-overlapping areas.

When multiple organs (e.g., ROI_A_, ROI_B_, and ROI_c_) overlap in the same projection. To estimate the true energy deposition for a specific target organ, the correction was performed in the following steps:

1. Identification of Multi-Overlap Pixels: The system uses a mask to identify pixels where the count of overlapping ROIs is ≥ 2.
2. Iterative Subtraction of Contributions: For the target organ (e.g., ROI_A_), the algorithm loops through every other overlapping ROIs (e.g., ROI_B_ and ROI_c_)
3. Mean-Based Estimation: For each overlapping ROI, calculates its mean energy deposition from its own non-overlapping regions.
4. Final Correction: The contributions of all other organs are subtracted from the total signal in the overlapped area:

$\sum_{i=1}^{n} A={\sum_{i=1}^{m} A}_{i,A-(A\cap B\cap C)}+{\sum_{i=1}^{n-m} (A}_{i,(A\cap B\cap C)}-{(\bar{B}}_{B-\left( A\cap B\cap C \right)}\times N_{A\cap B\cap C})-{(\bar{C}}_{C-\left( A\cap B\cap C \right)}\times N_{A\cap B\cap C}))$ (S2.2)

This framework accounts for that the energy deposition is not double-counted or triple-counted in anatomically crowded areas, such as where the liver, right kidney, and a nearby lesion might all overlap in a single planar projection. By using the mean of non-overlapping regions as a reference, the method provides a patient-specific estimation of the cross-dose contribution from each tissue layer.

**Validation of overlapping correction**

The OC method was validated using planar images from two scenarios involving overlapping spheres placed within a cylindrical water phantom, as shown in Fig.S2.2(a) for overlapping spheres of the same size and Fig.S2.2(b) for overlapping spheres of different sizes. The total ^177^Lu activity was 37 MBq. In both scenarios, two activity concentration conditions were evaluated: (1) equal activity concentration in both spheres and (2) one sphere having double the activity concentration of the other. Absorbed dose values for each sphere, calculated using the EPK method with OC, were directly compared to reference values obtained from MC simulations performed with PHITS software to assess accuracy.


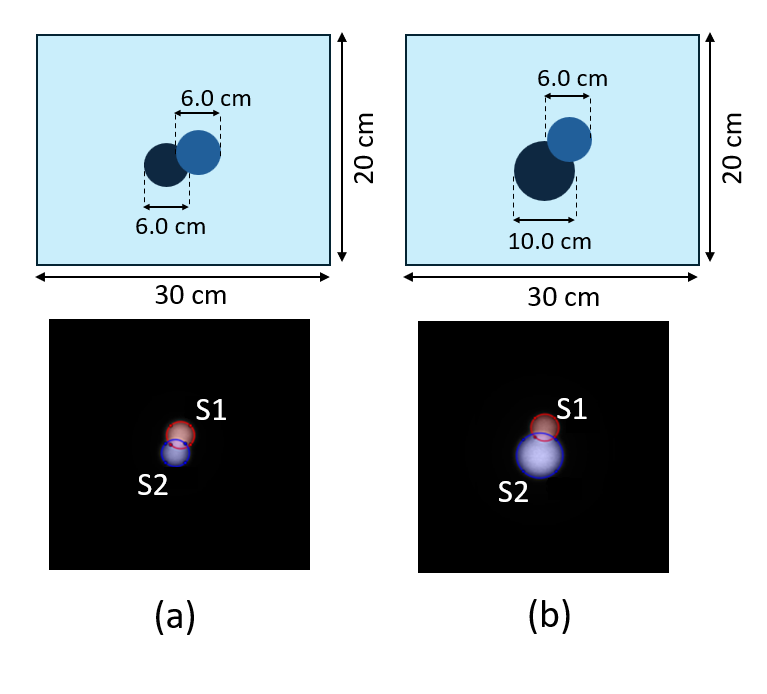


**FIGURE S2.2** Overlap spheres phantom and ROIs on energy deposition map of
(a)overlapping same-spheres size (b) overlapping different-spheres size

The result of validation of OC in absorbed dose by the EPK convolution method with overlapping spheres phantom showed that absorbed dose in each ROI was overestimated by compared to MC simulation, due to activity summation in overlapping region. After applying OC, estimation errors were significantly reduced, as shown in Table S1. The inaccuracy caused by overlapping regions was strongly influenced by both the extent of overlap and the size of the objects involved and also activity concentration. In cases involving spheres of different sizes, the overlap effect was more pronounced in smaller spheres, where activity from overlapping regions led to substantial overestimation. These results confirm that overlapping activity regions are a major source of error in planar dose estimation and point out the importance of implementing OC, especially when evaluating small or closely positioned anatomical structures.

**TABLE S2** Percentage difference of absorbed dose in spheres between EPK convolution applied OC and MC

simulation

| **Phantom conditions** | | | **ROIs** | **% difference of absorbed dose compares with MC simulation** | | |
| --- | --- | --- | --- | --- | --- | --- |
| **Spheres size** | | **Concentration*** |  | **EPK** | **EPK&OC** | |
| Same size  (The diameters of S1 and S2 are 6 cm.) | | 1:1 | S1 | 17.68 | 4.38 | |
|  |  |  | S2 | 17.79 | 4.62 | |
|  |  | 2:1 | S1 | 38.90 | 7.62 | |
|  |  |  | S2 | 6.94 | 2.79 | |
| Different size  (The diameters of S1 is 6cm and S2 is 10 cm.) | | 1:1 | S1 | 75.22 | 8.36 | |
|  |  |  | S2 | 16.48 | 7.25 | |
|  |  | 2:1 | S1 | 37.90 | 6.92 | |
|  |  |  | S2 | 26.88 | 9.76 | |
|  |  | 1:2 | S1 | 146.74 | 8.91 | |
|  |  |  | S2 | 10.92 | 5.68 | |
| * Ratio of activity concentration between sphere S1 and S2 | | | | |  |  |

**Reference**

1. He B, Frey EC. Comparison of conventional, model-based quantitative planar, and quantitative SPECT image processing methods for organ activity estimation using In-111 agents. Phys Med Biol. 2006;51(16):3967–3981. doi:10.1088/0031-9155/51/16/006.
