# Supplementary material for: Development of energy deposition pixel kernel convolution for planar dosimetry in 177Lu therapy
Source: Ann Nucl Med. 2026 Mar 16;40(7):814–22. doi: 10.1007/s12149-026-02191-3 (PMC13283131; doi:10.1007/s12149-026-02191-3)
Supplement: Supplementary file 3 — Supplementary Material 3 [file 12149_2026_2191_MOESM3_ESM.docx]

**Supplemental Data 3**

**TIA map generation**

- **Digital human phantom**

Pseudo voxel-based time-integrated activity (TIA) map [Bq·s/ml/voxel] was generated for the digital human phantom by assigning uniform TIA reference values from TIA data of ^177^Lu-DOTATAE Patient A of the open-access SNMMI Dosimetry Challenge [1] for both male and female phantom. These values were applied to all voxels of the liver, kidneys, spleen, and remaining body. To validate the method while excluding uncertainties associated with quantitative planar imaging, a pixel-based TIA map was generated by projecting the voxel-based TIA map. This projection resulted in partial overlaps between the liver and the right kidney, as well as between the liver and the spleen. Table S3.1 presents the estimated organ TIA values, derived by defining volumes of interest (VOI) on the voxel-based TIA map and projecting them to regions of interest (ROI) on the pixel-based TIA map, incorporating overlap correction.

**TABLE S3.1** Organ TIA of digital human phantom model estimated from voxel-based and pixel-based TIA maps

| **Organ** | **Organ TIA (Bq·s)** | | | |
| --- | --- | --- | --- | --- |
|  | **Male** | | **Female** | |
|  | Voxel-based | Pixel-based | Voxel-based | Pixel-based |
| Liver | 2.02E+14 | 2.08E+14 | 1.79E+14 | 1.83E+14 |
| Rt.Kidney | 2.41E+13 | 2.25E+13 | 1.76E+13 | 1.70E+13 |
| Lt.Kidney | 2.60E+13 | 2.63E+13 | 1.90E+13 | 1.92E+13 |
| Spleen | 3.31E+13 | 2.83E+13 | 2.97E+13 | 2.45E+13 |
| Remaining body | 6.96E+13 | 6.95E+13 | 4.74E+13 | 4.88E+13 |

- **Patient data**

Voxel-based TIA maps were generated from multiple SPECT time-points using trapezoidal integration, with physical decay applied for extrapolation after the last time-point [10]. This voxel-based TIA maps utilized SPECT/CT data from Patient A (male, 7.21 GBq) and Patient B (female, 7.31 GBq), who were imaged at 3.7, 27.7, 103.1, 124.0 h and at 3.7, 32.6, 99.6, 193.3 h, respectively [2]. To validate the method while excluding the uncertainties typically associated with quantitative planar imaging, pixel-based TIA map was generated by directly projecting the voxel-based TIA map. This projection created partial overlaps between the liver and the right kidney, as well as between the liver and the spleen. Table S3.2 presents the estimated organ TIA values, derived by defining volumes of interest (VOI) on the voxel-based map and projecting them to regions of interest (ROI) on the pixel-based TIA map with overlap correction.

**TABLE S3.2** Organ TIA of patient data estimated from voxel-based and pixel-based TIA maps

| **Organ** | **TIA (Bq·s)** | | | |
| --- | --- | --- | --- | --- |
|  | **Male** | | **Female** | |
|  | Voxel-based | Pixel-based | Voxel-based | Pixel-based |
| Liver | 2.17E+14 | 2.32E+14 | 1.12E+14 | 1.40E+14 |
| Rt.Kidney | 5.04E+13 | 4.90E+13 | 4.49E+13 | 4.22E+13 |
| Lt.Kidney | 5.28E+13 | 5.27E+13 | 1.74E+13 | 1.71E+13 |
| Spleen | 7.36E+13 | 7.37E+13 | - | - |
| Lesion1 | 1.38E+14 | 1.27E+14 | 2.66E+12 | 2.48E+12 |
| Lesion2 | 1.18E+13 | 1.12E+13 | 2.31E+13 | 2.12E+13 |
| Lesion3 | - | - | 7.69E+12 | 7.15E+12 |
| Lesion4 | - | - | 4.13E+12 | 4.21E+12 |
| Remaining body | 3.20E+14 | 3.15E+14 | 4.36E+14 | 4.15E+14 |

**Organ mass information**

Table S3.3 presents organ mass information for the ICRP 110 reference model (used in the MIRD method) and the Japanese adult phantom, alongside patient-specific data estimated from CT images.

**TABLE S3.3** Organ mass of phantom model and patients

| **Organ (g)** | **Male** | | | **Female** | | |
| --- | --- | --- | --- | --- | --- | --- |
|  | ICRP110  Adult [3] | Japanese adult phantom [4] | Patient A [2] | ICRP110  Adult [3] | Japanese adult phantom [4] | Patient B [2] |
| Liver | 1800.01 | 1284.10 | 2076.86 | 1400.00 | 1001.12 | 1639.93 |
| Rt.Kidney | 157.04 | 151.04 | 249.43 | 125.53 | 95.96 | 178.01 |
| Lt.Kidney | 153.00 | 152.32 | 239.01 | 149.48 | 103.94 | 84.06 |
| Spleen | 149.99 | 145.536 | 262.14 | 130.00 | 113.86 | - |
| **Body weight (kg)** | 73.00 | 63.00 | 92.99 | 60.00 | 53.00 | 51.71 |
| **Height (cm)** | 176.00 | 171.40 | 175.30 | 163.00 | 159.00 | 162.60 |

**Reference**

1. Dewaraja YK, Van BJ. Lu-177 DOTATATE anonymized patient datasets: Lu-177 SPECT time integrated activity maps. Deep Blue Data website Published August 23, 2021. Modified November 18, 2022. doi:10.7302/9nct-bk44. Accessed September 28, 2025.
2. Dewaraja YK, Van BJ. Lu-177 DOTATATE anonymized patient datasets: multi-time point Lu-177 SPECT/CT scans. Deep Blue Data. Published February 10, 2021. . Modified November 18, 2022. doi:10.7302/0n8e-rz46. Accessed September 28, 2025.
3. International Commission on Radiological Protection. (2009). Adult reference computational phantoms (ICRP Publication No. 110). Annals of the ICRP, 39(2).
4. Nagaoka T, Watanabe S, Sakurai K, Kunieda E, Watanabe S, Taki M, Yamanaka Y. Development of realistic high-resolution whole-body voxel models of Japanese adult males and females of average height and weight, and application of models to radio-frequency electromagnetic-field dosimetry. *Phys Med Biol*. 2004;49:1–15. doi:10.1088/0031-9155/49/1/001.
